# Insights into the sticking probability of volcanic ash particles from laboratory experiments

**DOI:** 10.1038/s41598-023-47712-6

**Published:** 2023-12-01

**Authors:** Carolina Diaz-Vecino, Eduardo Rossi, Stefano Pollastri, Allan Fries, Jonathan Lemus, Costanza Bonadonna

**Affiliations:** Département des Sciences de La Terre, Université de Genève, Geneva, Switzerland

**Keywords:** Environmental sciences, Volcanology

## Abstract

Although the characterization of the sticking and aggregation probability is essential to the description of volcanic ash dispersal and sedimentation, there is still no general model describing the sticking probability of volcanic ash. Experiments of dry particle–plate collisions in an enclosed box were carried out to characterize quantitatively the sticking efficiency of volcanic particles and silica beads in a limit case scenario where the mass of one of the particles is much greater than the others. Silica beads and volcanic particles from a Sakurajima Vulcanian eruption were filmed impacting a glass plate with a High-Speed Camera. The sticking probability is calculated from an equation depending on the particle diameter, impact velocity, and two experimental parameters (a, q). Particle size was found to dominantly control the sticking probability, with small particles more likely sticking on the glass plate than large particles. These experiments represent a significant step forward in the quantification of the sticking efficiency of fine volcanic ash (< 63 μm).

## Introduction

Particle aggregation has a high potential to impact the aerodynamic behavior and the residence time of volcanic ash in the atmosphere^1,2^. Volcanic Ash Transport and Dispersal Models (VATDMs) might overestimate the ash concentration in the far field and underestimate ground ash loading close to the source if ash aggregation is not accounted for^3–5^. Even though volcanic ash aggregation has been the focus of many field, experimental and numerical studies of the last few decades^2,3,6–14^, only a few VATDMs account for particle aggregation^15,16^, and even fewer can be used for operational forecasting^17^. Despite these recent advances, VATDMs still require additional refinement, notably in order to incorporate adequate descriptions of the mechanisms that favor aggregation, such as electrostatic forces or the occurrence of a liquid bonding^2,11,12^.

Aggregation models used in VATDMs require a better constrain of both collision and sticking efficiency^4,18,19^. The collision efficiency quantifies the probability of particles to collide with each other^4,20^, while the sticking efficiency is the probability that particles stick after a collision^21^. Both these parameters are used within the Smoluchowski Coagulation Equation (SCE)^22^ that describes the rate at which particles coagulate. The collision efficiency depends on the size and relative velocity of the colliding objects and can be significantly affected by electrical interactions, especially when fine particles carry significant electrostatic charges^23^. The determination of collision and sticking efficiency through laboratory experiments is a challenging problem that needs to be fully solved^24^. In fact, collision efficiency is more conveniently constrained with numerical simulations^24–26^. On the other hand, the sticking efficiency or sticking probability is better characterized based on an experimental approach^8,27–30^.

During the last decades, crucial experimental work was carried out to understand the role that sticking efficiency plays in the aggregation of volcanic particles^8,27,28,31^. However, most of the experiments on this subject focus on the qualitative description of the sticking efficiency for volcanic particles, only few of them provide quantitative estimations. For instance, Telling and Dufek^10^ performed a quantitative experiment using particles with diameters ranging from 90 to 250 µm to determine the relationship between the relative humidity, the collisional kinetic energy, and the aggregation efficiency. They used a pressurized gas line to accelerate the samples through a vertical nozzle and make particles collide with each other. As expected, the results showed that low relative collisional kinetic energy corresponds to high aggregation efficiency. Their work is a significant step towards a better understanding of the dynamics of volcanic particle aggregation. However, it is necessary to perform similar experiments using fine ash (< 63 μm) because this size fraction plays a fundamental role in particle aggregation^2^. In particular, we need to identify whether particles stick or rebound after a collision, and which is the threshold velocity below which rebound does not occur (hereafter named the *critical velocity*). The critical velocity can be rigorously defined as the maximum relative velocity of two colliding particles above which sticking does not occur. Two scenarios exist (wet and dry aggregation), which involve different dissipation mechanisms. For wet collisions, the energy is mainly dissipated by the viscous forces provided by the liquid layers present around the particles’ surface; for dry collisions, energy dissipation occurs due to Van Der Waals adhesion forces and by viscoelastic forces associated with particle deformation^28,32,33^.

The present work aims to characterize the sticking probability of particles with a diameter < 63 µm by analyzing particle–plate collisions to simulate a small particle colliding with a larger particle (i.e., a particle of infinite mass and radius). This type of collision occurs, for example, during the formation of coated particles (PC2) or cored clusters (PC3), where very fine ash particles of a few microns collide and stick with coarse ash particles of a few tens to a few hundred microns^2,12,34^. The particle–plate setting is a limit case scenario that allows us to create a preliminary model for the collision of particles of very different sizes. Using a high-speed camera, particle–plate collisions were recorded and later analyzed to measure the diameter and impact velocity of each colliding particle.

Intervals of velocity and diameter were selected to create a matrix of the probability of sticking (i.e., the ratio between the number of particles that successfully stuck on the plate and the total number of particles released) for both volcanic particles and silica beads (See methodology section). Ultimately, we found an empirical equation to describe the sticking probability as a function of the diameter and the impact velocity for both types of particles.

## Results

### Sticking probability matrix

**Figure 1:**
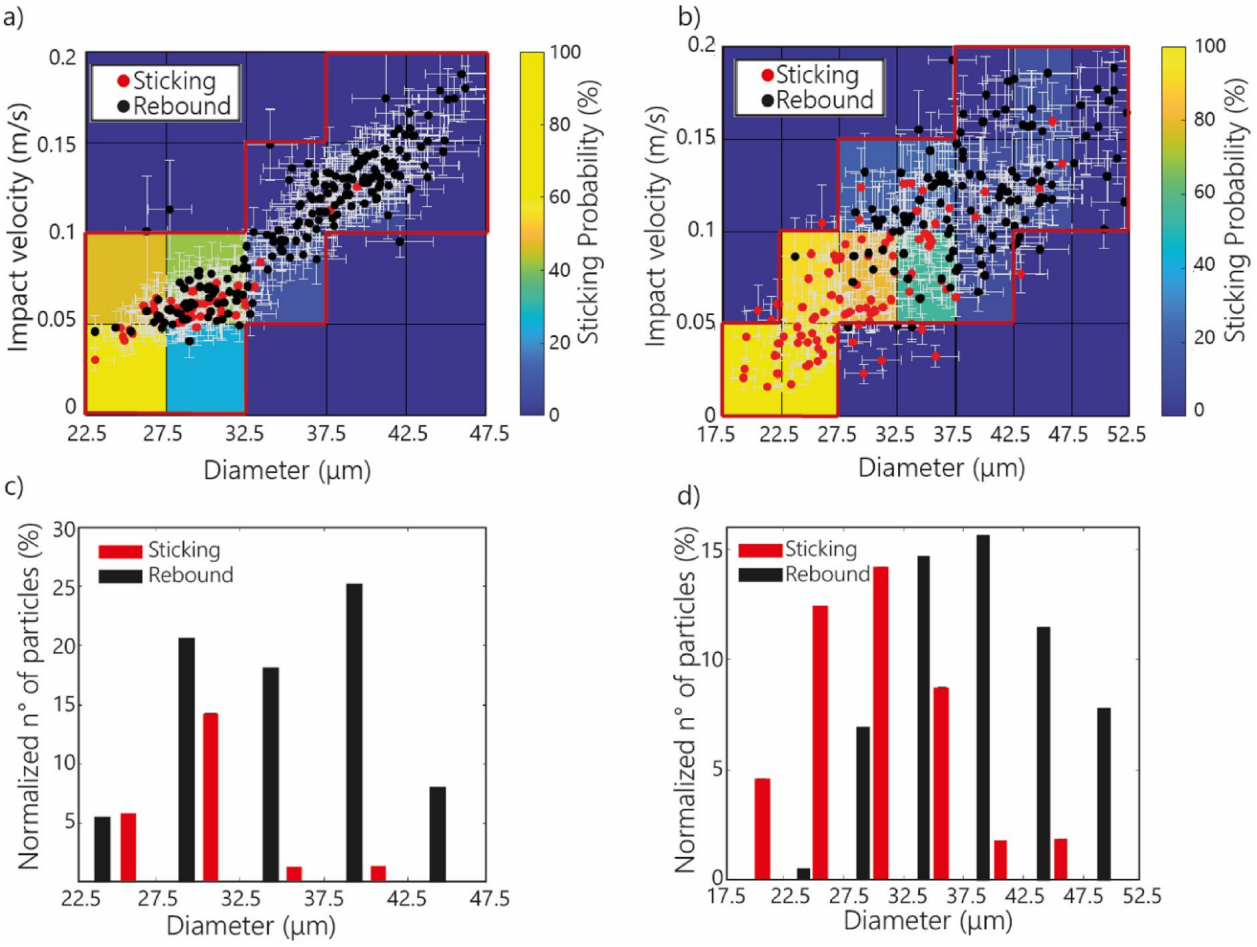
Impact velocity against particle diameter for (**a**) silica beads and (**b**) volcanic ash particles. Red and black circles represent sticking and rebound events, respectively. Red lines bound the data points considered in this study. The particle size distribution for the particles that stuck (in red) and that rebounded (in black) are shown for (**c**) silica beads and (**d**) volcanic ash particles.

The impact velocity of particles varies with diameter for both volcanic ash particles and silica beads (Fig. 1), with a greater scatter spread for volcanic ash particles (Fig. 1b) in comparison with spherical silica beads (Fig. 1a). Volcanic ash particles ranging from 19.26 to 52.28 μm and silica beads ranging from 23.03 to 46.17 μm were used in this study. The entire list of raw collision data can be found in Supplementary Tables S1 and S2. We selected the intervals for size (every 5 μm), and for velocity (every 0.05 m s^−1^) to have a significant number of particles within each matrix bin for the information to be representative (i.e., more than five particles). Five particles represent about 2% of the total collisions we filmed for both particle types (i.e., 236 for silica beads and 217 for volcanic particles). Although the size range for volcanic particles is wider than for silica beads, the size intervals remain the same (each 5 µm).

Results show that small particles are more likely to stick on the glass plate than large particles (Fig. 1). In particular, most small (diameter < 32.5 μm) volcanic ash particles (~ 84%) and about half of the small silica beads (~ 55%) stuck on the glass plate; about 25% and 5% of the medium-size (diameter comprised between 32.5 and 42.5 μm) volcanic ash particles and silica beads stuck on the plate, respectively; and none of the large (diameter > 42.5 μm) silica beads particles and only 5% of volcanic ash particles stuck with the plate (Fig. 1 and Supplementary Table S1).

### Sticking probability curve

Given the binary nature of the collision outcome (stick or rebound), we choose to describe the sticking probability with Eq. 1, which describes an S-shaped curve (see methodology).

1 $$P\left( {d_{p } ,v_{imp} } \right) = \frac{1}{{1 + \left( {aKd_{p}^{2} v_{imp} } \right)^{q} }}*100$$

This can be mathematically transformed into a sigmoid function, commonly used in logistic regression to describe binary outcome probabilities. However, this form is preferred as it explicitly demonstrates the dependence of sticking efficiency on both diameter and impact velocity. The convenience of this expression becomes evident when one observes that the sticking efficiency approaches zero as both the diameter ${d}_{p}$ and collision velocity ${v}_{imp}$ increase, and conversely, it approaches 100% as both these parameters tend towards zero.

The Stokes number describes the behavior of particles in a fluid, and it was used in this work as a mathematical tool to fit the diameter and impact velocity parameters (See methodology section). Besides them, Eq. (1) depends on three parameters: *q* characterizes the behavior of the curvature (i.e., the lower the value the flatter the curve); *a* represents a multiplicative constant in the equation; and* K* is a constant number from the Stokes number equation with a value of 1.52 × 10^8^ s m^−3^ (Table 1). The parameters *q* and *a* are calculated by best fitting experimental data with Eq. (1), whilst *K* is calculated from the density of the particles, the kinematic viscosity of air and a characteristic dimension (Eq. 4).

**Table 1:** Experimental parameters values found for Eq. (1) and the associated coefficient of determination (R-squared). The parameters a and q are found to best fit the data.

Parameter	Silica beads	Volcanic particles
*a*	223.40	61.73
*q*	1.00	2.77
*R-squared*	0.70	0.93

**Figure 2:**
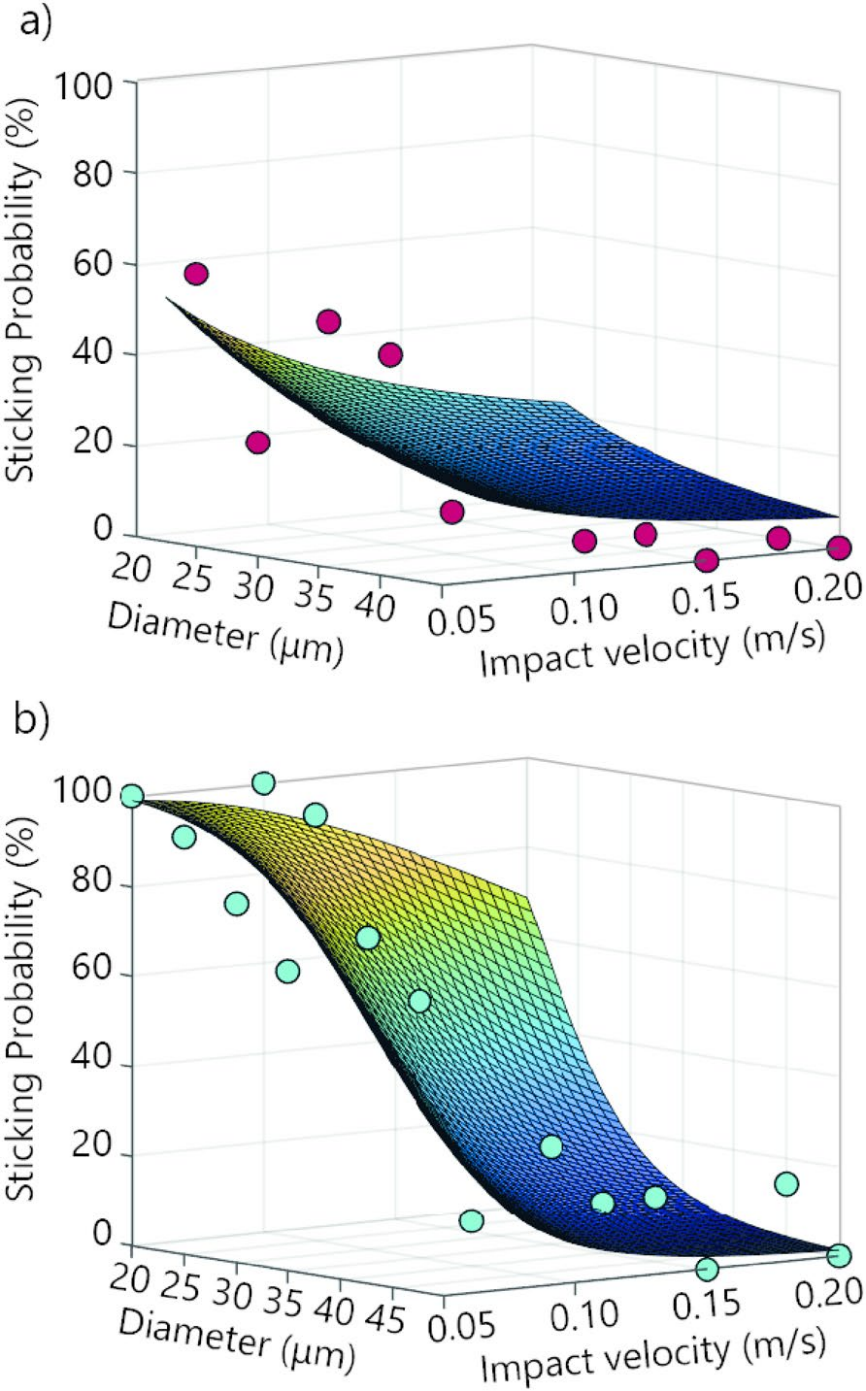
3D graphic representation of the sticking probability for (**a**) silica beads and (**b**) volcanic ash particles. The points represented in the graph are found using the midpoint values of each impact velocity and diameter interval as input for Eq. (1). The red points are associated with the silica beads and the light blue points are associated with volcanic ash particles. Additionally, the 3D curve is constructed to fit the points.

Using Eq. (1) for the mean value of each bin for the diameter (i.e., between 20 and 50 µm for volcanic particles and between 25 and 45 µm for silica beads) and the velocity (i.e., between 0.05 and 0.2 m s^−1^ for both particle types) (Fig. 1a, c), we constructed a graphic representation in 3D of the relationship between the size of the particles, their impact velocity, and their probability of sticking (Fig. 2). In Eq. (1), both the particle diameter and the velocity inversely control the sticking probability. Therefore, the greater the values of ${d}_{p}$ and ${v}_{imp}$, the lower the probability of sticking. However, as ${d}_{p}$ is squared, it influences the sticking probability more than the impact velocity, and the particle size predominantly controls the sticking with the plate.

Another way to reveal the influence of particle size over the impact velocity is by analyzing Eq. (1) in more depth. If we fix the velocity at 0.05 m s^−1^ and change the diameters from 20 to 50 μm, we obtain a decrease in the sticking probability of 60% for volcanic ash particles and 54% for silica beads (Table 2). Moreover, if we fix the velocity at 0.2 m s^−1^, the probability decrease is 66% and 21% for volcanic ash particles and silica beads, respectively. On the other hand, if we fix the diameter to 20 μm and change the velocity from 0.05 to 0.2 m s^−1^, we find that the probability decrease is 30% for volcanic particles and 32% for silica beads. Finally, if we fix the diameter to 50 μm and apply the same procedure, the probability decreases by 37% for volcanic particles and 13.5% for silica balls. These results show that the chance of sticking is more severely affected by a variation in the diameter than in the velocity.

**Table 2:** Variation of sticking probability for different values of diameters and impact velocities based on Eq. (1), for both volcanic ash particles and silica beads.

Volcanic ash particles			Silica beads		
Diameter (μm)	Velocity (m s^−1^)	Probability (%)	Diameter (μm)	Velocity (m s^−1^)	Probability (%)
Variation of the particle diameter					
20	0.05	99.03	20	0.05	59.56
30		91.56	30		39.56
40		68.79	40		26.91
50		39.03	50		19.07
20	0.20	68.79	20	0.20	26.91
30		18.91	30		14.06
40		4.52	40		8.43
50		1.35	50		5.56
Variation of the impact velocity					
20	0.05	99.03	20	0.05	59.56
	0.10	93.76		0.10	42.41
	0.15	83.02		0.15	32.93
	0.20	68.79		0.20	26.91
50	0.05	39.03	50	0.05	19.07
	0.10	8.58		0.10	10.54
	0.15	2.96		0.15	7.28
	0.20	1.35		0.20	5.56

## Discussion

For the first time, particle collision and sticking have been analyzed for particles < 63 μm, which represent the main size fraction found in volcanic aggregates^12,35,36^. Impact velocities were varied by simply modifying the drop height release during experiments. However, for the same height, the impact velocity on the glass plate can vary depending on secondary particle properties such as shape, projected area and density. For both volcanic ash particles and silica beads, our results show that small particles (< 32.5 μm) did not reach velocities higher than 0.13 m s^−1^. The lowest sticking velocity value for volcanic ash particles is 0.016 m s^−1^ for a diameter of 21 μm. In contrast, the lowest sticking velocity for silica beads is 0.030 m s^−1^ for a diameter of 23 μm. As previously mentioned, the difference in the settling velocity for a given size is mostly due to the particle's shape (i.e., the drag coefficient). Indeed, the drag force acting on a sphere will be lower than for an irregular shape. Thus, the sphere will settle faster^37^. Various experimental approaches were tested to obtain higher velocities, like using a particle disperser; however, with this methodology the velocities of the particles were extremely high and obtaining an adequate video with this condition was a challenge. Moreover, the acceleration of particles is not the best approach for our study as the size range used within the experiments is very low, which means that the Stokes number of the particles is also low. Consequently, particles would not collide but would follow the fluid flow. On the other hand, large particles (> 42.5 μm) accelerated faster than small ones, and it was not possible to obtain impact velocities lower than 0.10 m s^−1^. Limitations with the determination of velocities include: (1) velocities smaller than 0.10 m s^−1^ were unachievable for particles larger than 42.5 μm for silica beads and 47.5 μm for volcanic ash particles; (2) velocities > 0.16 m s^−1^ were unachievable for particles smaller than 32 μm for both types. Additionally, even though volcanic particles are irregular in shape, we calculate their diameters using Eq. (9) from Bagheri et al.^38^ that provides an average error of 5.5%, which is acceptable, and a significant improvement compared to the average error of 26% found extrapolating irregular shapes into a circle. Regardless of these limitations, the amount of data gathered in this experimental work is sufficient to constrain the sticking probability.

We found that sticking probability is primarily influenced by particle size. Particles of diameters < 32.5 µm were more likely to stick on the glass plate due to the lower impact velocities (Fig. 1). For collisions characterized by lower impact velocities, the impact kinetic energy is lower, and therefore, collision energy is completely dissipated during impact resulting in the particle adhering to the plate. In contrast, larger particles with higher impact velocities have enough impact kinetic energy to bounce after the collision. Theoretically, collision outcome could be predicted comparing the collision velocity with the critical sticking velocity (which can be computed from the size of the colliding particle and its mechanical parameters). The critical sticking velocity correspond to the maximum relative velocity that two sticking particles may have beyond which the sticking does not occur anymore^39^. However, our findings indicate that while the probability of sticking does indeed decrease at higher velocities, there is not a distinct critical velocity for each size that determines whether particles stick or bounce. The transition is rather gradual. In fact, the change between a sticking probability of 100% (all sticking) and a sticking probability of 0% (all rebound) happens over a range of sticking velocities. This highlights the role played by various other factors (e.g., shape, surface roughness, electrical interactions) on adhesion forces during contact with the glass surface. The probability of sticking for volcanic ash particles is higher than for silica beads for the same size range (Fig. 1). This can result from the fact that volcanic particles have irregular shapes that are associated with higher number of contact points with the glass plate than for spherical silica beads, which allow them to stick more easily by increasing the contact surface and, thus, adhesion forces.

The sticking probability defined in Eq. (1) is found experimentally for volcanic ash and silica beads. The 3D fitting curve is expressed in terms of the Stokes number of the particle, here simply used as a mathematical support that embodies both the impact velocity and the diameter of the particles, which are the two main essential parameters to model particle aggregation. It is thus important to clarify that using the Stokes Number to reach the final equation was for practical reasons and fitting purposes. Nonetheless, the Stokes number has been used previously to characterize the aggregation efficiency of colliding particles in wet and dry collisions^4,33^. For instance, Costa et al.^4^ use the viscous Stokes number to derive a formula for aggregation efficiency. Their approach considers particles sticking in the presence of liquid water or ice, neglecting electrostatic aggregation, using the experimental results of Gilbert and Lane^27^ to parametrize the model.

In our case, Eq. (1) is experimentally derived under the specific conditions and assumptions reported in the paper. For instance, this equation may be applied for all those cases where particle sticking occurs in the absence of a liquid layer (e.g., electrostatic and adhesion forces in the aggregation process). In addition, the parametrization based on the particle–plate experiments results in different coefficients $a$ and $q$ for the two different particle types (i.e., volcanic particles and silica beads) within the equation; the notation of each parameter in the manuscript is presented in Table 3. Finally, the equation fits better for the volcanic ash particle experiments due to the challenge of obtaining low-impact velocities for silica beads. However, although not in perfect agreement, the R-squared for the silica beads curve is 0.7, which is acceptable to fit the sticking efficiency.

**Table 3:** Notation list for the parameters mentioned in the manuscript.

$A$	Area of the circle
$a$	Empirical multiplicative constant
$d$	Distance over which the ruler stayed in focus
$d_{c}$	Plate characteristic dimension
$d_{circle}$	Diameter of the circle
$d_{eq}$	Equivalent diameter
$d_{p}$	Particle’s diameter
$D_{i}$	Diameter of the largest inscribed circle
$D_{c}$	Diameter of the smallest inscribed circle
$DOF$	Depth of field
$f$	Frate rate (in fps)
$f_{i}$	Frame in $i$
$K$	Constant derived from the Stokes number; $K=p_\rho/18 \mu d_p$
$P$	Sticking probability
$P_{CKE}$	Sticking probability as a function of the Collision Kinetic Energy
$q$	Empirical constant
$St$	Stokes number
$v_{xi}$	Impact velocity in x
$v_{yi}$	Impact velocity in y
$v_{zi }$	Impact velocity in z
$v_{total} = v_{Imp}$	Total impact velocity
${\Delta }t$	Duration elapsed between two successive frames
${\Delta }T$	Duration elapsed between the first and the last frames
$\varphi_{Riley}$	Riley circularity
$\mu$	Viscosity of the air
$\rho_{p}$	Density of the particle

**Figure 3:**
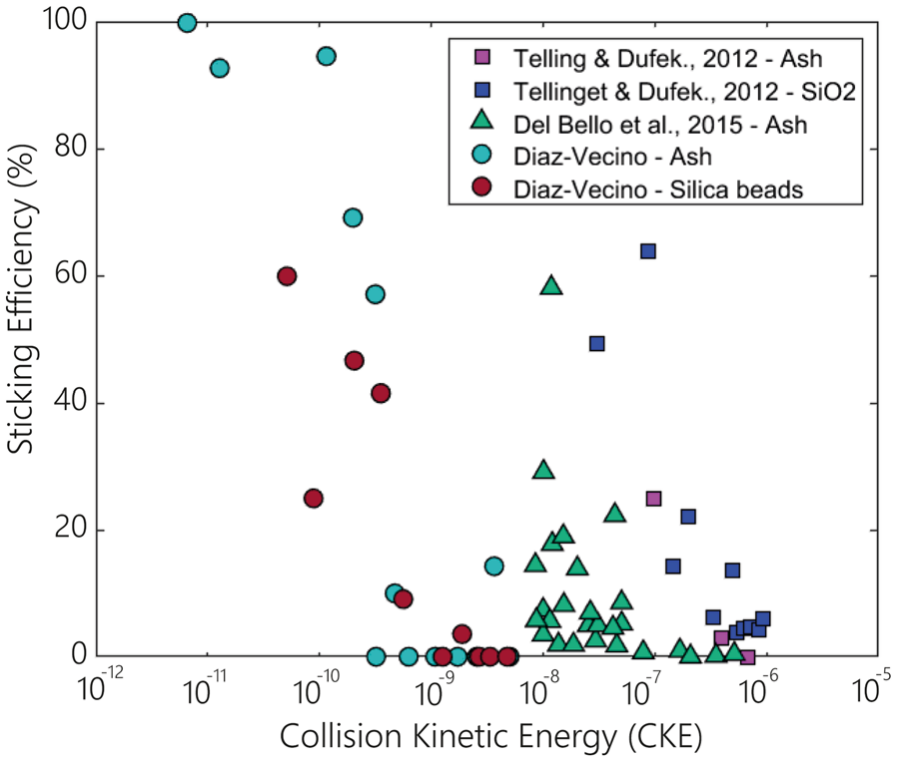
Variation in the sticking efficiency as a function of the collision kinetic energy for both volcanic particles and silica beads. The present study is represented by circles, whilst the results of Telling and Dufek^10^ and Del Bello et al.^31^ are represented by squares and triangles, respectively. These studies are based on particle–particle interactions for particles > 90 µm (i.e., Telling and Dufek^10^) and < 90 µm (i.e., Del Bello et al.^31^), while this study is focused on particle–plate interactions with particles < 63 µm. All the experiments were performed with variable relative humidity values and without liquid layers involved.

A common way to describe the collisional energy of particles is to calculate the collision kinetic energy (CKE). Telling and Dufek^10^ and Del Bello et al.^31^ provide a relationship between the CKE and the aggregation efficiency (Fig. 3). Contrarily to our experiments, these two studies involved different experimental configuration and focused on particle–particle collisions. The trend between these studies in comparison with our study is similar yet shifted to the right (i.e., higher kinetic energy). The particle sizes studied by Telling and Dufek^10^ range between 90 and 150 μm and reflect different humidities (20–80%); despite the higher kinetic energy in the collisions (i.e., ${10}^{-7}$ mJ), they observed sticking efficiencies up to 60%, even though most collisions had efficiencies below 20%. Their results suggest collisions had higher sticking efficiencies than expected from our results. While this could theoretically be due to higher humidity in their experiments, Telling and Dufek^10^ did not find a significant correlation between the presence of higher values of relative humidity and a more effective sticking efficiency. Therefore, a plausible explanation could be that their particles were highly charged due to numerous collisions in particle-laden jets, increasing the stickiness of their collisions. On the other hand, Del Bello et al.^31^ show a CKE between ${10}^{-6}$ and ${10}^{-8}$ mJ for particles < 90 µm and for a relative humidity of 50–60%. Their data is shifted to the left (i.e., lower CKE for equivalent sticking efficiencies) compared to Telling and Dufek^10^, which is associated with their experiments' disaggregation effects and turbulence. Our results show the lowest CKE (i.e., ${10}^{-8}$ to ${10}^{-11}$) for particles < 63 µm with a relative humidity of 20–30% without measurement of particle charges. Given that particles were released individually in our experiments, it is nonetheless expected that their charge is lower than in the configurations of Telling and Dufek^10^ and Del Bello et al.^31^. Hence, the result of this investigation effectively show that our experiments setup represents a limit case scenario in nature, where other mechanisms can facilitate aggregation (i.e., humidity, electrostatic forces).

**Figure 4:**
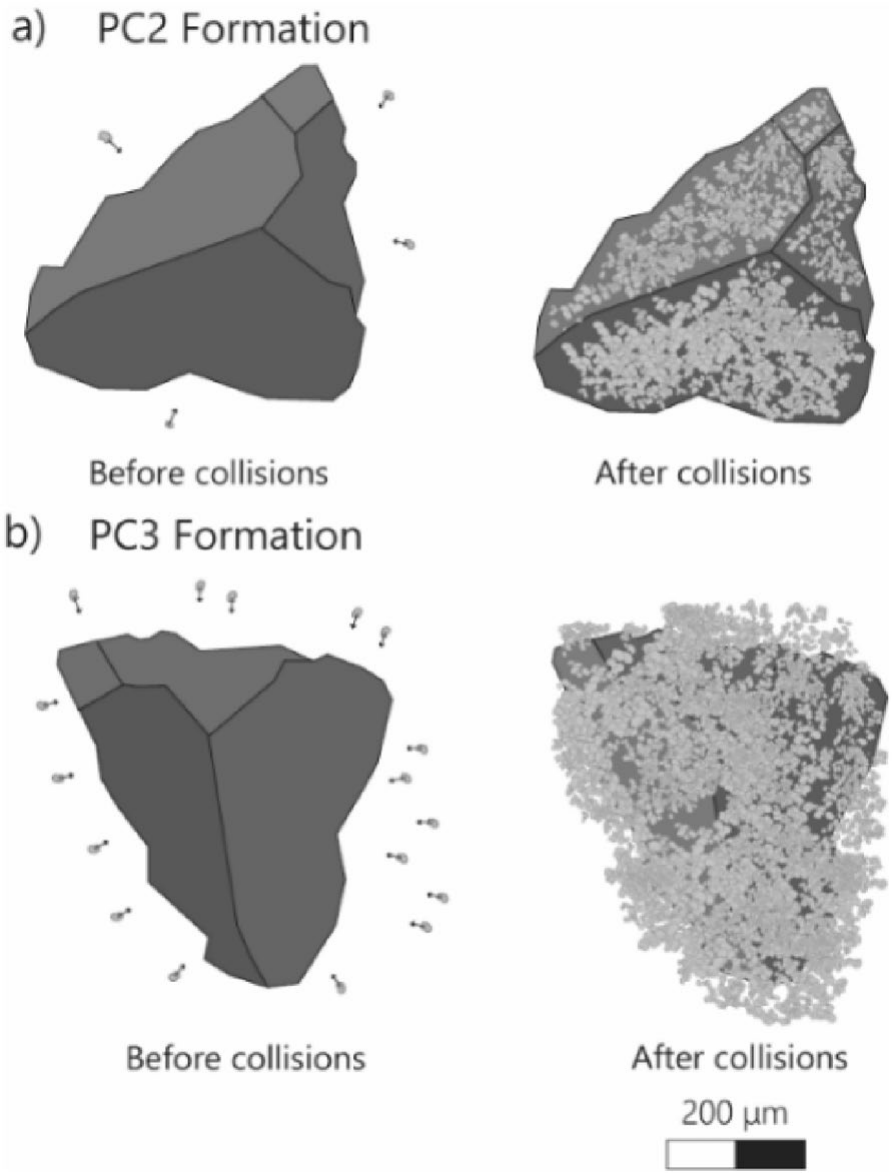
Sketch illustrating the formation of two types of Particle Clusters (PCs). (**a**) Coated particle (PC2) and (**b**) Cored cluster (PC3), before and after collisions with smaller particles. This sketch represents the natural process of formation for two types of aggregates where small particles collide with a significantly bigger one. This image is modified from Bagheri et al.^12^.

The results of this work can also be related to the interaction between small particles with diameters < 10 µm and coarse particles with diameters up to a few hundreds of µm. In fact, our experiments represent a case scenario where the mass of one of the particles is much greater than the others, as it is the case of aggregates known as coated particles (PC2) and cored clusters (PC3) as shown in Fig. 4. These types of aggregates can be present in both dry and wet eruptions^36^, therefore, the relationship derived from our experiments (dry conditions), is applicable. Accordingly, this work's outcome is a simplified approach for models trying to understand aggregation related to the formation of PC2 and PC3 only. Equation (1) quantifies a process that is challenging to measure during natural volcanic eruptions. However, limited by the controlled laboratory environment (i.e., no liquid bond, smooth contact surfaces, no external factors like wind, temperature, turbulence, etc.), our results provide a framework where forces are less dissipated than in a volcanological context. In fact, the occurrence of liquid bonding between particles in volcanic clouds, or the presence of highly charged volcanic ash particles, can increase the sticking efficiency^23^. As a result, for the same parameters (${d}_{p },{v}_{imp}$ ), the sticking probability is expected to be higher in nature than in our experiments. The results presented in this work therefore yield the minimum sticking efficiency of fine particles involved in the formation of PC2 and PC3 aggregates. They can be used as a first approximation to estimate the minimum collision velocity and size that can result in sticking in the case of a real volcanic cloud.

## Conclusions

Particle–plate experiments represent the first important step to better constrain the sticking probability of fine volcanic ash, which means particles with a diameter < 63 μm. We obtained a sticking probability equation, described by Eq. (1), based on experimental work for volcanic ash particles and silica beads. This equation is a function of the particles' velocity and diameter. We observed that sticking is mainly influenced by particle size in our experimental setup, which means that the smallest particles are more likely to stick, as they have low kinetic energy at collision available for rebound. Equation (1) constitutes a helpful tool for modeling aggregation as it comprises two important and easy-to-deduce parameters of particles: velocity and size. Additionally, the experimental setting and results can be related to the formation of particle clusters PC2 and PC3, which involve the collision of fine ash particles with a much coarser particle. This study provides key insights into sticking probability for fine ash that can be further developed by varying the experimental conditions, including particle–particle collisions, wet aggregation, controlled particle charge, changing temperature. Additionally, further work can also include running experiments with a turbulent fluid phase and higher particle concentration to enhance the probability of particle collision and sticking.

## Methods

### Experimental set-up

**Figure 5:**
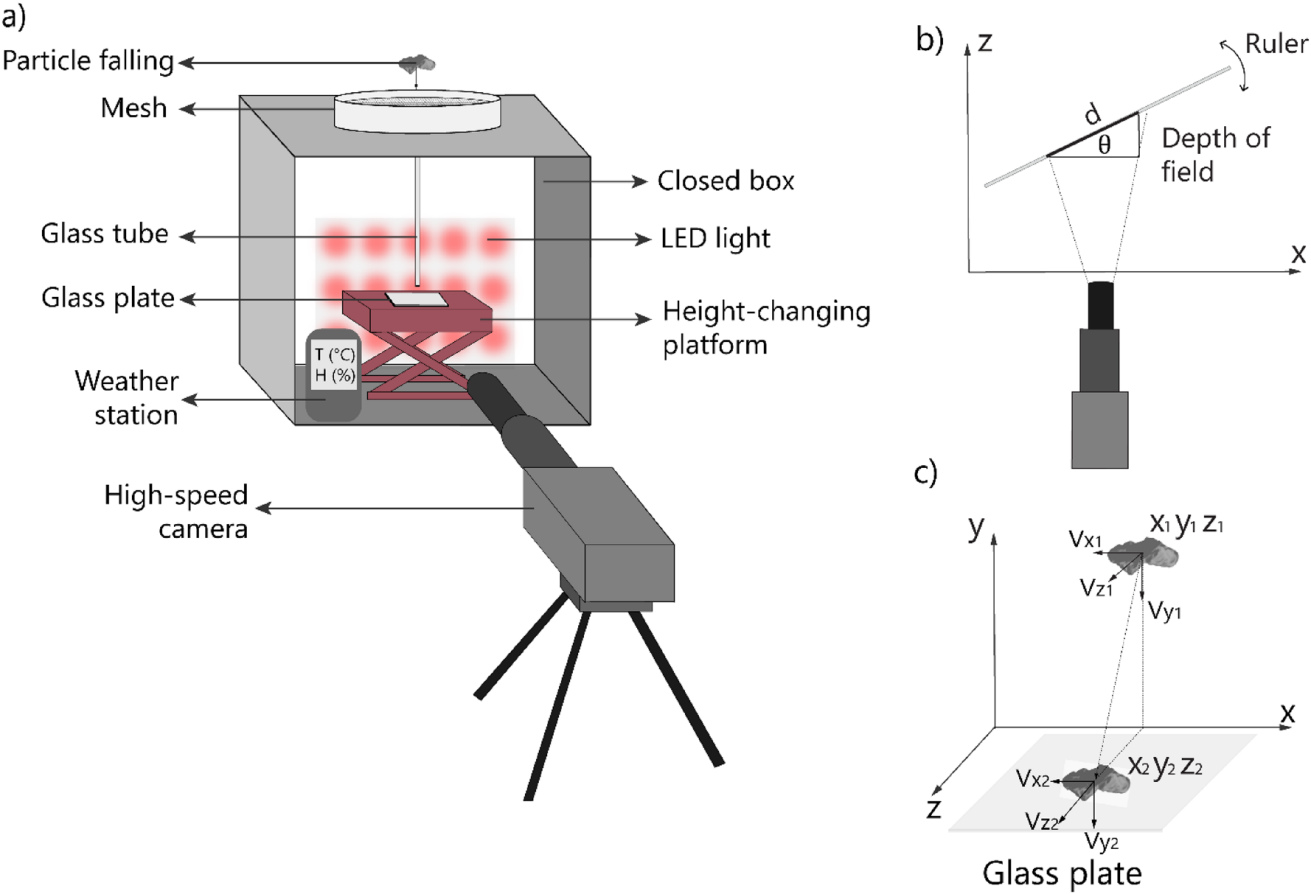
(**a**) Sketch of the particle–plate set-up where the high-speed camera records the particle plate collisions. (**b**) Scheme measuring the depth of field (DOF), where d and $\uptheta$ are known parameters. By moving the ruler along the z-axis, we could detect the distance the camera stayed on focus (**c**) Particle falling to the glass plate: ${\mathrm{v}}_{\mathrm{xi}}$ and ${\mathrm{v}}_{\mathrm{yi}}$ are measured directly with the position; velocity ${\mathrm{v}}_{\mathrm{zi}}$ is inferred using the depth of field (DOF). The maximum value for ${\mathrm{v}}_{\mathrm{zi}}$ is obtained when a particle goes through the whole depth of field, maintaining focus.

Prior to the experiments, particles were sieved down to three size classes: < 32 μm, 32–45 μm, and 45–63 μm. Two types of particles were used in separate experiments, including glass beads (i.e., spherical silica beads), and volcanic ash particles collected at Sakurajima volcano (Japan) during a Vulcanian eruption occurred on 31 July 2013^12^. During the experiments, particles were released onto a glass plate from different heights by placing them in a sieve that was gently tapped to generate a soft vibration that allowed single particles to pass through (Fig. 5a). Different sieve mesh sizes (32 μm, 45 μm, 63 μm) were used for particles of different diameters. After the release, the particles descended through a vertical glass tube before colliding with the glass plate. The release height used in these experiments was set at 5, 10, 15, 20, 25, and 30 cm above the glass plate to obtain a wide range of impact velocities. The collisions were filmed using a Phantom M110 High-Speed Camera (HSC) with a long-distance lens tc16m009 from Opto-engineering, located perpendicular to the glass plate, and a closed glass box was placed surrounding the set-up to prevent the impact of external factors such as air currents (Fig. 5a). Moreover, A LED light and an optical diffuser were located behind the glass box to guarantee the best light conditions to highlight particle collisions, and a Kestrel 5500 weather meter was located next to the setting to measure the temperature, humidity, and air velocity after every experiment, confirming that no significant variations of any of these environmental parameters was detected in between experiments.

### Depth of field

The depth of field (DOF) is the distance of acceptable sharpness and focus on a picture or video. A ruler was slowly moved in front of the camera to detect the distance $d$ for which the graduations were in focus (Fig. 5b). The DOF was calculated as the ratio between $d$ and the measured angle ${\uptheta }$ as $DOF = d*{\text{sin}}\left( {\uptheta } \right),$ with a value of 60 μm for this experimental setting.

### Measurement of the velocity

The instantaneous velocity along the *x* and *y* axes (defined on the plane of the camera recording) was measured by tracing particles manually across frames. Therefore, the velocity was found using the change in position and the shift in time from the first frame $$f_{i}$$ to the following one $$f_{i + 1}$$. The duration $${\Delta }t$$ elapsed between successive image frames is given by $${\Delta }t = 1/f$$, with $$f$$ the frame rate (ranging from 4000 to 6000 fps $$)$$. Therefore, the instantaneous velocity of a particle in *x* can be approximated by $$v_{xi} = (x_{i + 1} - x_{i} )/{\Delta }t$$, with $$x_{i}$$ and $$x_{i + 1}$$ the horizontal position of the particle in frames $$i$$ and $$i + 1$$, respectively. The same methodology applies for $$v_{yi}$$, the instantaneous velocity in the y direction. Particles were analyzed carefully, so all particles were in focus all the time while falling onto the glass plate. Hence, the velocity in the z coordinate $$v_{zi}$$ should ideally be zero, but it is possible that the particle moves in the *z* direction, even if it is in focus (Fig. 5c). In this case, the best approximation of the maximum velocity in the *z* direction is the ratio between the DOF and the time difference between the first and the last frame obtained $${\Delta T}$$, so that $$v_{zi } < \left| {v_{zi} } \right|_{max} = DOF/{\Delta T}$$. The total velocity before the collision $$v_{total}$$ is obtained using the three components of the velocity between the two frames before the impact with the glass plate. It is therefore constrained by the following inequality, following the Pythagorean Theorem for two and three dimensions

2 $$\sqrt {v_{xi }^{2} + v_{yi }^{2} } \le v_{total} \le \sqrt {v_{xi }^{2} + v_{yi }^{2} + v_{zi }^{2} } .$$

In this work that aims at determining a statistically reliable upper bound for the sticking velocity, the maximum value was used to estimate the impact velocity against the glass plate $v_{imp}$ $\left( {{\text{i}}.{\text{e}}.,v_{imp} \approx \sqrt {v_{xi }^{2} + v_{yi }^{2} + v_{zi }^{2} } } \right).$

### Measurement of the diameter

Silica beads are almost perfectly spherical, with very few irregularities in their shape; their diameter is therefore well constrained by that of a circle $d_{circle} = \sqrt {4A/\pi }$ with an area $A$ equal to that of the particle measured in all frames. The method of Bagheri et al.^38^ was used to estimate the size and shape of irregular volcanic ash particles from empirical correlations. The equation for the equivalent diameter ($d_{eq}$) in two dimensions is given by

3 $$d_{eq} = \frac{{{\text{max }}\left( {d_{circle} } \right)}}{{1.119\left( { \varphi_{Riley}^{ - 0.37} } \right)}},$$

where $d_{circle}$ is calculated using the maximum value of all the areas measured frame by frame, the $\varphi_{Riley}$ is the Riley circularity^40^ expressed as $\varphi_{Riley} = \sqrt {D_{i} /D_{c} }$ where $D_{i}$ and $D_{c}$ are the diameters of the largest and smallest inscribed circle, respectively.

## Analytical method

### Probability matrix and change of variables approach

During each experiment, individual volcanic particles and silica beads collided with the plate, causing one of the observed processes: (1) sticking or (2) rebounding. In total, 236 silica beads were analysed; 50 beads stuck on the glass plate, and 186 rebounded after impact. On the other hand, a total of 217 collisions were studied for volcanic ash particles, including 93 particles that stuck and 124 particles that rebounded. Using the previous information (i.e., impact velocity, diameter, rebounding or sticking), we constructed a sticking probability matrix (Fig. 1), dividing the graph into intervals for impact velocity (every 0.05 m s^−1^) and diameter (every 5 μm). These intervals were defined to have a significant number of points for the information to be representative (more than 5 points). Therefore, the information was not considered when there were fewer points in a given interval. Consequently, only the information enclosed in the red line in Fig. 1 is used in this study. The probability represents the number of particles sticking with respect to the total amount of particles in each interval of velocity and diameter. Thus, a probability value of 100% means that all the particles stuck, while a value of 0% means that all the particles rebounded. For silica beads, the maximum probability reaches up to 60%, whereas for volcanic particles it is up to 100%.

To combine the most critical parameters of this study (i.e., diameter and velocity) in a single equation, we use the Stokes number, which describes the behavior of particles in a fluid as shown in Eq. (4). The Stokes number has been used previously to characterize the aggregation efficiency of colliding particles in wet and dry collisions^4,33^. The link results from the fact that particles with more inertia have higher Stokes numbers and are less likely to aggregate^33^. Therefore, the Stokes number is considered a suitable criterion for studying the particle sticking probability. In addition, it also represents a mathematical tool to fit the previously mentioned parameters. In this study, we derive the Stokes number $St$^41^ per each interval of particle size and velocity and compare it to the sticking probability $P$ per bin. Therefore, we used the following equation:

4 $$St = Kd_{p}^{2} v_{imp} ,$$

where $d_{p}$ is the diameter of the particle, $v_{imp}$ is the velocity of the particle, *K* = $\rho_{p} /18 \mu d_{c}$, with $\rho_{p}$ the particle density, $d_{c}$ = 0.05 m is a characteristic dimension (here the length of the plate), and $\mu$ = 1.85 × ${10}^{{ - 5}}$ kg m^−1^ s^−1^ is the dynamic viscosity of the air from Gottlieb and Ritzel^42^ for 25 °C, which was the average temperature in the laboratory during the experiments. The particle density $\rho_{p}$ was set at 2500 kg m^−3^ based on measurements by Bagheri et al.^12^ on volcanic ash particles of Sakurajima volcano for the same period. As input for $d_{p}$ and $v_{imp}$ we used the mid-value for each interval in the constructed probability matrix. For instance, the first diameter taken for volcanic particles and silica beads was 20 μm and 25 μm, respectively; and the first value of velocity taken for both particles was 0.025 m s^−1^.

Using the MATLAB tool *cftool* to fit the points from the Stokes number vs. Sticking probability curve, we found the following relationship:

5 $$P\left( {d_{p } ,v_{imp} } \right) = \frac{100}{{1 + \left( {a St} \right)^{q} }} ,$$

The equation was obtained after trying multiple S-shaped curves to describe our data, including exponential and trigonometric functions. As the Stokes number is a function of the impact velocity and the diameter, it is simple to obtain the probability of sticking P as a function of $d_{p }$ and $v_{imp}$ (Eqs. 1 and 5). The empirical parameters $a$ and $q$ derived from fitting are different for volcanic ash and silica beads.

### Supplementary Information

Supplementary Table S1.

Supplementary Table S2.

Supplementary Movie S1.

Supplementary Movie S2.

## Data Availability

All data generated or analyzed during this study are included in this published article and its supplementary information files.
