# Supplementary material for: Insights into the sticking probability of volcanic ash particles from laboratory experiments
Source: Sci Rep. 2023 Dec 1;13:21188. doi: 10.1038/s41598-023-47712-6 (PMC10692354; doi:10.1038/s41598-023-47712-6)
Supplement: Supplementary file 1 — Supplementary Table S1. [file 41598_2023_47712_MOESM1_ESM.docx]

**Supplementary information for: Insights into the sticking probability of volcanic ash particles from laboratory experiments**

Carolina Diaz-Vecino^1^; Eduardo Rossi^1^; Stefano Pollastri^1^; Allan Fries^1^; Jonathan Lemus^1^; Costanza Bonadonna^1^

^1^ Département des Sciences de la Terre, Université de Genève, Geneva, Switzerland

[carolinadiazvecino@gmail.com](mailto:carolinadiazvecino@gmail.com)

1. **Probability matrix**

| ***Silica beads*** | | | ***Volcanic particles*** | | |
| --- | --- | --- | --- | --- | --- |
| Interval of $d_{p}$  (μm) | Interval of $v_{imp}$  (m s^-1^) | P  (%) | Interval of $d_{p}$  (μm) | Interval of $v_{imp}$  (m s^-1^) | P  (%) |
| 22.5 -27.5 | 0-0.05 | 60.0 | 17.5-22.5 | 0-0.05 | 100.0 |
| 22.5 -27.5 | 0.05-0.1 | 46.6 | 22.5-27.5 | 0-0.05 | 92.8 |
| 27.5 -32.5 | 0-0.05 | 25.0 | 22.5-27.5 | 0.05-0.1 | 94.7 |
| 27.5 -32.5 | 0.05-0.1 | 41.5 | 27.5-32.5 | 0.05-0.1 | 69.2 |
| 32.5 -37.5 | 0.05-0.1 | 9.1 | 27.5-32.5 | 0.1-0.15 | 18.7 |
| 32.5 -37.5 | 0.1-0.15 | 0.0 | 32.5-37.5 | 0.05-0.1 | 57.1 |
| 37.5-42.5 | 0.1-0.15 | 3.5 | 32.5-37.5 | 0.1-0.15 | 21.7 |
| 37.5-42.5 | 0.15-0.2 | 0.0 | 37.5-42.5 | 0.05-0.1 | 10.0 |
| 42.5-47.5 | 0.1-0.15 | 0.0 | 37.5-42.5 | 0.1-0.15 | 11.1 |
| 42.5-47.5 | 0.15-0.2 | 0.0 | 37.5-42.5 | 0.15-0.2 | 0.0 |
| - | - | - | 42.5-47.5 | 0.1-0.15 | 14.2 |
| - | - | - | 42.5-47.5 | 0.15-0.2 | 14.2 |
| - | - | - | 47.5-52.5 | 0.1-0.15 | 0.0 |
| - | - | - | 47.5-52.5 | 0.15-0.2 | 0.0 |

**Table S1.** Values for the sticking probability $P$ for each interval of particle diameter $d_{p}$ and impact velocity $v_{imp}$for volcanic particles and silica beads. The values presented in this table correspond to the information bounded by the red line in Fig. 1a,b.

1. **Uncertainties analysis**

*Uncertainty on the impact velocity*

The modulus of the linear velocity is the greater value of the inequality given by Eq (3): $v_{total}= \sqrt{{v_{xi}}^{2} +{v_{yi}}^{2} +{v_{zmaxi}}^{2}}$ and is calculated using the velocities along the $x$, $y$and $z$ axes. The first step towards obtaining the uncertainty $\delta v_{total}$ is to calculate the uncertainties of the velocities in the three components ($\delta v_{xi}, \delta v_{yi} and \delta v_{z maxi})$. To do so, we used the linearized propagation of errors for mutually independent variables. The general formula for the propagation of error states that given the measurements$x_{1},x_{2},.., x_{n}$ with uncertainties $\delta x_{1},{\delta x}_{2},.., \delta x_{n}$and given the function $w= f (x_{1},x_{2},.., x_{n}$), the uncertainty in $w$ is

| $\delta w=\sqrt{\left( \frac{\partial w}{\partial x_{1}} \delta x_{1} \right)^{2}+ ...+ \left( \frac{\partial w}{\partial x_{n}} \delta x_{n} \right)^{2}}$ | Eq. S1 |
| --- | --- |

We use$v_{xi}= {(x}_{i+1}-x_{i})/\Delta t$ and $v_{yi}= {(y}_{i+1}-y_{i})/\Delta t$ to find $\delta v_{xi}, \delta v_{yi}$ and ${v_{zi}< \left| v_{zi} \right|}_{max}=DOF/\Delta T$ to find $\delta v_{z max}$. As mentioned in the methodology section, to find velocities $v_{xi}$ and $v_{yi}$, we analyzed the images frame by frame from the videos, and to find $v_{z maxi}$ we assumed the value of the depth of field. In that sense, the way of calculating the uncertainties for $\delta xi$, $\delta yi$ is different than for $\delta v_{z maxi}$.

We need to remember that $v_{xi}= f(x_{i+1}, x_{i}, t_{i+1}, t_{i})$ and $v_{yi}= f(y_{i+1}, y_{i}, t_{i+1}, t_{i})$, therefore we are going to obtain uncertainties for the distance and time. However, the uncertainty of time is given by the chronometer of the camera and is linked to its precision, usually this value is very low and for these experimental measurements is negligible. When we apply Eq (S1) for finding, for example, the uncertainty $\delta v_{xi}$ we obtain the following expression:

| $\delta v_{xi}=\sqrt{\left( \frac{\partial v_{xi}}{\partial x_{i+1}} \delta x_{i+1} \right)^{2}+ \left( \frac{\partial v_{xi}}{\partial x_{i}} \delta x_{i} \right)^{2}}$ | Eq. S2 |
| --- | --- |

Solving the derivatives, we obtain the following expression

| $\delta v_{xi}=\sqrt{\left( \frac{1}{\Delta t} \delta x_{i+1} \right)^{2}+ \left( -\frac{1}{\Delta t}\delta x_{i} \right)^{2}}$ | Eq. S3 |
| --- | --- |

Given that the uncertainties $\delta x_{i+1}$ and $\delta x_{\text{i}}$ have the same value that is calculated considering the error due to pixel length and is 4.92 µm for all the videos, Eq (S3) can be written as:

| $\delta v_{xi}= \frac{\delta x}{\Delta t} \sqrt{2}$ | Eq. S4 |
| --- | --- |

The same procedure applies for finding $\delta v_{yi}$ and we obtain a similar equation

| $\delta v_{yi}= \frac{\delta y}{\Delta t} \sqrt{2}$ | Eq. S5 |
| --- | --- |

However, finding the uncertainty $\delta v_{zmaxi}$ is different because we are using the $DOF$ as the distance to calculate the velocity, so we need to consider the uncertainty in the $DOF$ measurement. The value calculated for the depth of field is 60 µm and we are going to assume an error of the 50% therefore the $\delta v_{zmaxi}$is 30 µm for all the particles. The error of 50% is the maximum error a particle could have as we only chose particles that were falling within the $DOF$. After finding the individual uncertainty for each velocity we apply again the general formula for the propagation of error for${v_{zi}< \left| v_{zi} \right|}_{max}=DOF/\Delta T$ and we obtained the following expression:

| $\delta v_{imp\_total} = \sqrt{\left( \frac{\partial v_{i}}{\partial v_{xi}} \delta v_{xi} \right)^{2} +\left( \frac{\partial v_{i}}{\partial v_{y}} \delta v_{yi} \right)^{2} +\left( \frac{\partial v_{i}}{\partial v_{zmaxi}} \delta v_{zmaxi} \right)^{2}}$ | Eq. S6 |
| --- | --- |

If we solve the derivatives and replaced the uncertainties $\text{δ}{\text{v}_{\text{x}}}^{\text{2}}\text{, }$ $\delta{v_{y}}^{2}$ and $\delta{v_{z}}^{2}$, we obtain the final expression to calculate the absolute error on the total impact velocity

| $\delta v_{imp\_total\_absolute} = \frac{1}{v_{i}}\sqrt{{v_{xi}}^{2}\delta{v_{xi}}^{2} +{v_{yi}}^{2}\delta{v_{yi}}^{2}+{v_{zmaxi}}^{2}\delta{v_{zmaxi}}^{2}}$ | Eq. S7 |
| --- | --- |

Hence, the relative error on the total impact velocity is

| $\delta v_{imp\_total\_relative} = \frac{1}{{v_{i}}^{2}}\sqrt{{v_{xi}}^{2}\delta{v_{xi}}^{2} +{v_{yi}}^{2}\delta{v_{yi}}^{2}+{v_{zmaxi}}^{2}\delta{v_{zmaxi}}^{2}}$ | Eq. S8 |
| --- | --- |

For the volcanic particles, the minimum relative error was 0.0319, and the maximum relative error was 0.31. On the other hand, for the silica beads, the minimum and maximum relative error was 0.07 and 0.337, respectively.

*Uncertainty on the diameter*

To obtain the uncertainty on the diameter we considered the formula used to calculate the diameter given by Eq (4). As mentioned earlier the calculations were made following the work of Bagheri et al., (2015). The author proposed the equation for finding the diameter of irregular particles in 2D as well as the associated relative error using empirical data. The formula used in this work corresponds to Eq (9) of Bagheri et al. (2015) and the relative error calculated by the author has a value of 5.5%.

1. **Movies**

The videos were taken with a resolution of 256x800 pixels, a frame rate of 40 frames per second, and a pixel size approximately of 5 µm.

**Movie S1.** Video of silica beads. Two particles of 38 and 28 µm rebound with the glass plate, and two particles of 23 and 28 µm stick with the glass plate.

**MovieS2.** Video of volcanic particles. Two particles of 32 and 49 µm rebound with the glass plate, and two particles of 25 and 40 µm stick with the glass plate.

**References**

1. Bagheri, G. H., Bonadonna, C., Manzella, I., & Vonlanthen, P. (2015). On the characterization of size and shape of irregular particles. *Powder Technology*, *270*, 141-153.
